# Misalignment Between Patient-Reported Quality of Life and Guideline-Based Preventive Care Indicators in People Living With HIV: A Cross-Sectional Study

**DOI:** 10.7759/cureus.107362

**Published:** 2026-04-19

**Authors:** Merhary B Meza-Matus, Cecilia Salud-Gutierrez, Nallely Rincón Peregrino, Elena López-Pineda, Alfredo González-Ayala, Quitzia L Torres-Salazar

**Affiliations:** 1 Family Medicine, Hospital General de Zona No. 2 con Medicina Familiar, Instituto Mexicano del Seguro Social, Salina Cruz, MEX; 2 Biomedical Sciences, Facultad de Medicina y Nutrición, Universidad Juárez del Estado de Durango, Durango, MEX

**Keywords:** healthcare quality indicators, heath related quality of life, hiv, patient-reported outcomes, preventive care

## Abstract

Background

The widespread use of antiretroviral therapy has transformed HIV infection into a chronic, manageable condition, shifting the focus of care toward patient-centered outcomes such as health-related quality of life (HRQoL). However, the extent to which HRQoL aligns with guideline-based healthcare quality indicators remains unclear.

Objective

To evaluate HRQoL and its alignment with guideline-based preventive care indicators in people living with HIV in a public healthcare setting.

Methods

An observational, cross-sectional study was conducted at a public secondary-care hospital of the Instituto Mexicano del Seguro Social (IMSS) in Oaxaca, Mexico, including 80 adult patients with HIV receiving outpatient care. Participants were selected through consecutive sampling. Inclusion criteria comprised adults (≥18 years) with a confirmed diagnosis of HIV infection under active follow-up, while patients with incomplete clinical records were excluded. HRQoL was assessed as a continuous variable using the Spanish version of the Medical Outcomes Study Short Form-30 (MOS-SF-30). Compliance with guideline-based preventive care indicators derived from Grupo de Estudio del SIDA (GESIDA) was calculated as the proportion of fulfilled indicators per patient. Descriptive statistics were used, and the relationship between variables was evaluated using Spearman’s rank correlation coefficient (rho).

Results

The median HRQoL score was 91 (q25-q75: 86.2-94). In contrast, overall compliance with GESIDA indicators was low, with a median of 28.5% (q25-q75: 22-33%). Antiretroviral therapy coverage was 100% (80/80 patients), whereas several preventive indicators, including hepatitis A vaccination and substance use screening, showed no compliance (0%). Preventive care indicators consistently showed lower performance compared to treatment-related indicators. No statistically significant correlation was observed between HRQoL and compliance with GESIDA indicators (rho = 0.014, p = 0.899).

Conclusions

People living with HIV exhibited high HRQoL despite low compliance with guideline-based preventive care indicators. The absence of correlation between these variables suggests a misalignment between objective measures of healthcare quality and patient-reported outcomes. These findings highlight the need to strengthen preventive strategies and adopt comprehensive, patient-centered approaches to optimize long-term care.

## Introduction

Human immunodeficiency virus (HIV) infection has undergone a profound epidemiological transformation over the past decades, with an estimated 39.9 million people living with HIV worldwide in 2023 and approximately 630,000 deaths attributed to HIV-related causes [1]. The widespread implementation of antiretroviral therapy (ART) has led to a 69% reduction in HIV-related mortality since its peak in 2004, effectively converting HIV from a nearly universally fatal disease into a chronic, manageable condition [2]. As life expectancy among people living with HIV approaches that of the general population, the focus of care has shifted beyond virological suppression and immunological recovery toward broader patient-centered outcomes, particularly health-related quality of life (HRQoL) [3].

HRQoL has emerged as a key indicator of treatment success, as it captures the multidimensional impact of HIV on physical, psychological, and social well-being, domains that are not fully reflected by traditional clinical markers such as CD4 count or viral load [4]. Concurrently, clinical practice has evolved toward the implementation of structured models of care that incorporate preventive, diagnostic, and longitudinal follow-up strategies. In this context, guideline-based indicators (such as those proposed by the Grupo de Estudio del SIDA [GESIDA]) provide a standardized and measurable framework to evaluate the quality of care delivered to people living with HIV. These indicators encompass key domains of care, including prevention of opportunistic infections, vaccination, screening for comorbidities, and long-term follow-up, and are designed to monitor healthcare performance and identify areas for improvement [5].

Despite these advances, the extent to which objective measures of healthcare quality align with patient-reported outcomes remains uncertain. This potential misalignment is clinically relevant, as HRQoL in people living with HIV is influenced not only by virological control but also by a complex interplay of psychosocial, behavioral, and structural determinants, including comorbidities, mental health conditions, and social support [4]. As life expectancy increases, non-AIDS-related conditions such as cardiovascular disease, metabolic disorders, and psychological distress have emerged as key contributors to overall well-being, which may not be adequately captured by clinical performance indicators alone. In this context, quality-of-care indicators such as those proposed by GESIDA are designed to measure, evaluate, and standardize excellence in HIV care based on evidence-based practices; however, their focus on process and clinical outcomes may not fully reflect the multidimensional nature of patient-perceived health [5]. Therefore, understanding the degree of alignment between these structured indicators and HRQoL is essential to ensure that healthcare models effectively address the comprehensive needs of this population. In this context, the present study aimed to evaluate the level of HRQoL and to explore its alignment with guideline-based preventive care indicators in people living with HIV treated in a public healthcare setting in Mexico.

## Materials and methods

An observational, cross-sectional, analytical study was conducted at the General Hospital of Zone with Family Medicine No. 2 “Francisco J. Macín Domínguez,” Instituto Mexicano del Seguro Social (IMSS), located in Oaxaca, Mexico. This is a public, social security-based healthcare institution that provides care to insured workers and their families, predominantly representing a middle- to low-income population in a high-demand clinical setting. The study was performed using data collected from patients attending the outpatient HIV clinic during the study period.

A total of 80 adult patients with a confirmed diagnosis of HIV infection under active outpatient follow-up were included. Participants were selected through a non-probabilistic consecutive sampling approach. Inclusion criteria comprised patients aged ≥18 years with a confirmed diagnosis of HIV infection and at least one clinical evaluation during the study period. Patients with incomplete clinical records or those who declined participation were excluded.

The primary outcome was HRQoL, measured as a continuous variable using the Spanish version of the Medical Outcomes Study Short Form-30 (MOS-SF-30), originally derived from the Medical Outcomes Study developed by Wu et al. [6] and later validated in Spanish-speaking populations by Remor. This questionnaire assesses multiple domains, including physical functioning, mental health, pain, energy/fatigue, and social functioning, generating a global score ranging from 0 to 100, where higher values indicate better perceived quality of life [7,8]. The instrument was used in accordance with its original validation and citation requirements.

The main exposure variable was the level of compliance with guideline-based preventive care indicators derived from GESIDA, expressed as the proportion of fulfilled indicators relative to the total number of applicable indicators per patient [9]. These standardized, evidence-based indicators evaluate key domains of HIV care, including preventive strategies, vaccination, screening for comorbidities, and long-term follow-up, providing measurable benchmarks for healthcare performance. Sociodemographic and clinical variables, including age, sex, and relevant clinical characteristics, were also considered.

To minimize information bias, standardized instruments were used for both HRQoL assessment and evaluation of healthcare quality indicators. Data were primarily obtained from institutional clinical records and systematically reviewed during extraction. In addition, selected indicators were corroborated through direct patient interviews at the time of data collection to enhance data accuracy. Participants were included consecutively during the study period to reduce potential investigator-driven selection bias.

The sample size was calculated using the formula for proportions in finite populations, assuming a 95% confidence level (Z = 1.96), an expected proportion of low quality of life of 0.57, an absolute precision of 0.09, and a source population of 257 patients under follow-up, resulting in a minimum required sample size of 80 participants. A non-probabilistic consecutive sampling approach was used, including eligible patients until the required sample size was reached.

Descriptive statistics were used to summarize the data. Continuous variables were expressed as median and interquartile range (q25-q75), given the distribution of the data, while categorical variables were reported as frequencies and percentages. The relationship between HRQoL and compliance with GESIDA indicators was evaluated using Spearman’s rank correlation coefficient (rho), given the non-normal distribution of the variables. A p-value <0.05 was considered statistically significant. Statistical analyses were performed using standard statistical software.

The study protocol was reviewed and approved by the corresponding institutional research and ethics committees of the IMSS (approval number: R-2024-2001-047). The study was conducted in accordance with the principles of the Declaration of Helsinki and national regulations for health research. Patients were initially identified through institutional clinical records; subsequently, data were collected using standardized data collection forms assigned with unique study codes, without including any personal identifiers. Only coded, de-identified data were accessible to the research team throughout the study, ensuring full protection of patient confidentiality.

## Results

A total of 80 patients with HIV were included in the analysis. The median age was 40 years (q25-q75: 32-53), and the majority were male (69, 86.3%). Most participants were single (60, 75.0%). Regarding clinical status, 70 patients (87.5%) were asymptomatic, while six (7.5%) and four (5.0%) were classified as symptomatic and AIDS, respectively. The predominant route of transmission was sexual contact among men who have sex with men (MSM) (47, 58.8%), followed by unknown (22, 27.5%) and heterosexual contact (11, 13.8%). More than half of the patients (46, 57.5%) had an undetectable viral load (<20 copies/mL), and seven (8.8%) presented complications, defined as the presence of HIV- or ART-related clinical events. The median time since diagnosis was eight years (q25-q75: 6-9.75), which was similar to the median duration of ART (Table 1).

**Table 1:** General Characteristics of the Study Population (n = 80) *Values are presented as median (q25–q75) unless otherwise indicated. AIDS: acquired immunodeficiency syndrome.

Variable	Value
Age (years)*	40 (32–53)
Sex, n (%)	
• Female	11 (13.8%)
• Male	69 (86.3%)
Marital status, n (%)	
• Single	60 (75.0%)
• Married	11 (13.8%)
• Cohabiting	6 (7.5%)
• Widowed	3 (3.8%)
Clinical stage, n (%)	
• Asymptomatic	70 (87.5%)
• Symptomatic	6 (7.5%)
• AIDS	4 (5%)
Route of transmission, n (%)	
• Heterosexual contact (vaginal intercourse)	11 (13.8%)
• Sexual contact among men who have sex with men (MSM)	47 (58.8%)
• Unknown	22 (27.5%)
Viral load, n (%)	
• Undetectable	46 (57.5%)
• <20 copies/mL	13 (16.3%)
• 20–40 copies/mL	12 (15.0%)
• >100,000 copies/mL	9 (11.3%)
Complications, n (%)	
• Yes	7 (8.8%)
• No	73 (91.3%)
Time since diagnosis (years)*	8 (6–9.75)
Time on antiretroviral therapy (years)*	8 (6–9.75)

Compliance with individual GESIDA indicators showed marked variability across domains (Table 2). The highest levels of compliance were observed in indicators related to treatment and initial clinical evaluation, including ART coverage (80, 100%), completeness of initial clinical history (44, 55.0%), and loss to follow-up monitoring (40, 50.0%). In contrast, indicators related to preventive care consistently showed lower compliance.

**Table 2:** Compliance With Individual GESIDA Indicators (n = 80) MSM: men who have sex with men; ART: antiretroviral therapy; STI: sexually transmitted infection; GESIDA: Grupo de Estudio del SIDA

GESIDA Indicator	n (%)
Late diagnosis of HIV infection in specialized care	38 (47.5%)
Relevant contents in initial clinical history	44 (55.0%)
Relevant serological testing at initial evaluation	37 (46.3%)
Patients receiving antiretroviral therapy	80 (100.0%)
Regular patient follow-up	32 (40.0%)
Latent tuberculosis infection screening	13 (16.3%)
Hepatitis A vaccination	0 (0.0%)
Hepatitis B vaccination	23 (28.7%)
Pneumococcal vaccination	13 (16.3%)
Human papillomavirus vaccination	7 (8.8%)
Smoking prevention and treatment	0 (0.0%)
Chemsex screening in MSM	12 (15.0%)
Screening for active drug use	0 (0.0%)
Syphilis screening	29 (36.3%)
Screening for other STIs in MSM	11 (13.8%)
Loss to follow-up	40 (50.0%)
Initiation of ART after first visit	25 (31.3%)
Undetectable viral load at week 48	12 (15.0%)
Adherence assessment	15 (18.8%)
Cervical cancer screening	8 (10.0%)
Hepatitis C treatment	9 (11.3%)
Cardiovascular risk assessment	10 (12.5%)
Metabolic syndrome screening	21 (26.3%)

Vaccination against hepatitis A (0%), smoking prevention and treatment (0%), and screening for substance use (0%) showed no documented compliance. Low compliance rates were also observed for latent tuberculosis screening (13, 16.3%), pneumococcal vaccination (13, 16.3%), and human papillomavirus (HPV) vaccination (7, 8.8%). Indicators related to initial assessment and treatment showed higher compliance rates than those related to prevention and comorbidity management.

The evaluation of HRQoL revealed a high median MOS-SF-30 score of 91 (q25-q75: 86.2-94), whereas the overall compliance with GESIDA indicators had a median of 28.5% (q25-q75: 22-33%) (Table 3).

**Table 3:** Global Evaluation of GESIDA Compliance and Health-Related Quality of Life (n = 80) Values are presented as median (q25–q75). MOS-SF-30: Medical Outcomes Study Short Form-30; GESIDA: Grupo de Estudio del SIDA.

Variable	Value, median (q25–q75)
MOS-SF-30 score	91 (86.2–94)
Global GESIDA compliance	28.5% (22%–33%)

**Figure 1:**
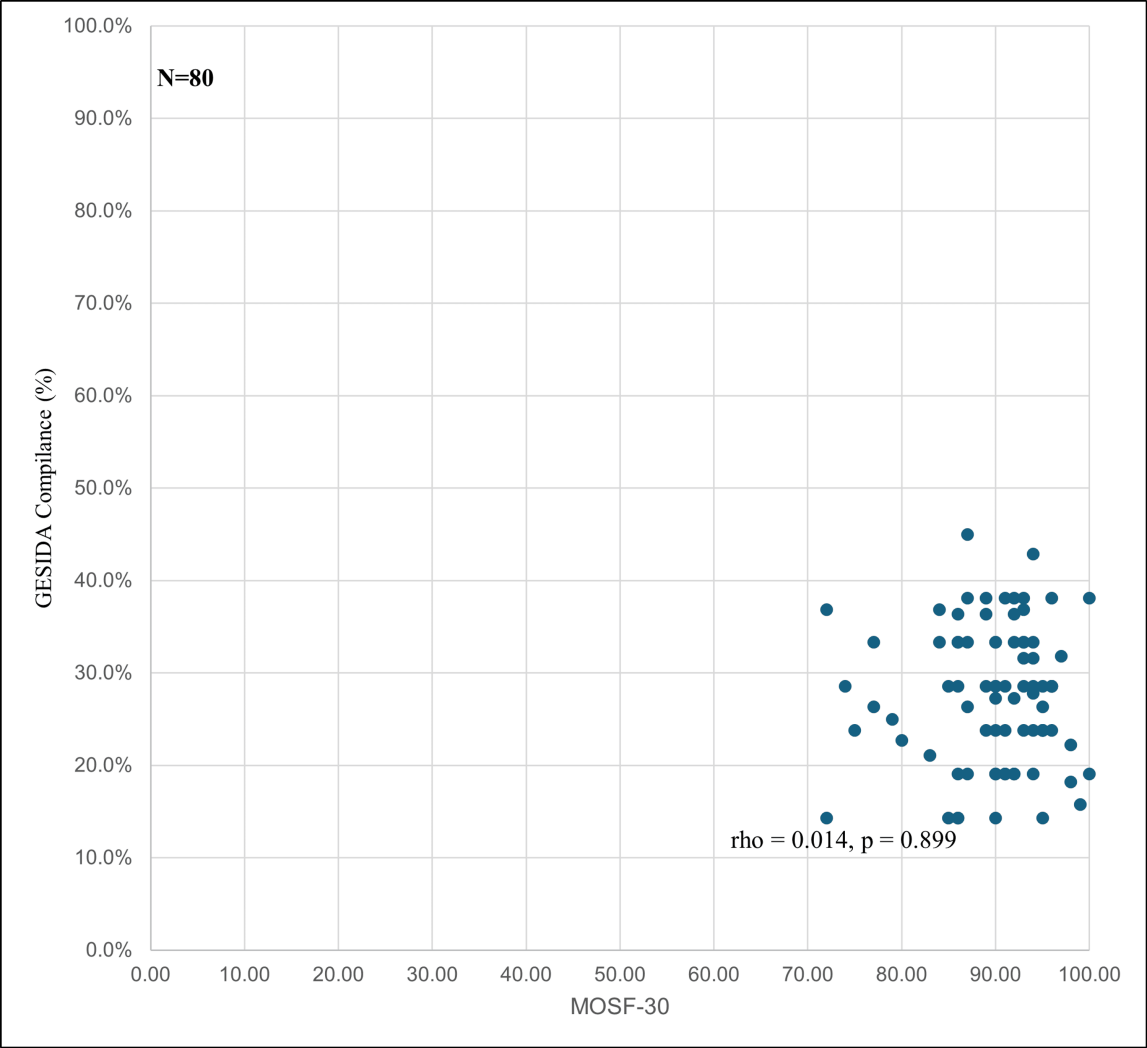
Relationship Between Health-Related Quality of Life (HRQoL) and Compliance With GESIDA Indicators in People Living With HIV No significant correlation was observed between HRQoL and compliance with GESIDA indicators (rho = 0.014, p = 0.899). MOS-SF-30: Medical Outcomes Study Short Form-30; GESIDA: Grupo de Estudio del SIDA.

Despite this disparity, no statistically significant correlation was observed between HRQoL and compliance with GESIDA indicators (rho = 0.014, p = 0.899), as illustrated in Figure 1.

## Discussion

The present study evaluated the relationship between HRQoL and compliance with guideline-based preventive care indicators in people living with HIV in a public healthcare setting. The findings revealed a misalignment between these dimensions: despite a high median HRQoL score, overall compliance with GESIDA indicators was lower, and no significant correlation was observed between the two variables. This pattern reflected differential performance across domains, with high compliance in treatment-related indicators and lower performance in preventive and comorbidity-related measures. These results suggest that patient-perceived well-being does not depend linearly on the degree of adherence to structured healthcare quality indicators, but rather reflects a more complex interaction of clinical stability, psychosocial factors, and contextual conditions.

The sociodemographic profile of the study population, characterized by a predominance of male patients, a median age of 40 years, and a high proportion of single individuals, is consistent with the epidemiological patterns of HIV reported in Latin America [10]. These characteristics are relevant when interpreting HRQoL, as factors such as social support, emotional adaptation, and acceptance of the diagnosis have been shown to significantly influence perceived well-being [11]. In this context, the absence of a stable partner or formal support network may not necessarily translate into lower quality of life, particularly in the presence of adaptive coping mechanisms and functional social integration.

From a clinical perspective, the high proportion of asymptomatic patients and the substantial percentage of individuals with undetectable viral load reflect adequate immunovirological control in a large segment of the sample. This clinical stability likely plays a central role in sustaining high HRQoL scores, as viral suppression is associated with reduced symptom burden, improved functional capacity, and overall better health perception [12]. Previous studies have consistently demonstrated that adherence to ART and favorable immunological status are strongly associated with improved quality of life, even in settings with structural limitations in healthcare delivery [13].

A key contribution of this study lies in the domain-based analysis of GESIDA indicators, which revealed a consistent pattern in healthcare performance. Indicators related to initial assessment and treatment showed higher levels of compliance, whereas those associated with preventive care and comorbidity management demonstrated lower performance. This distribution suggests a differential emphasis within the current model of care, which appears to be more strongly oriented toward diagnosis and pharmacological control, with comparatively less integration of preventive strategies and long-term risk management. These findings provide a structured framework to identify potential gaps in care delivery beyond individual indicators.

In particular, preventive care indicators (such as vaccination, screening for latent tuberculosis, and interventions targeting modifiable risk factors) showed minimal or null compliance, representing the lowest-performing domain. In this study, compliance with these indicators reflects the implementation and documentation of healthcare processes rather than patient-level behavior. These findings should be interpreted within the context of real-world clinical practice, where the availability, prioritization, and systematic implementation of certain preventive interventions may vary across healthcare settings. This finding highlights a critical gap in the comprehensive management of people living with HIV. While these deficiencies may not immediately impact perceived quality of life, they represent a significant risk for future morbidity and underscore the need for strengthening preventive and health promotion strategies within HIV care programs [14].

This pattern is consistent with broader global challenges in the HIV response, where advances in treatment and reductions in mortality have not been matched by equivalent progress in prevention. Fragmented, vertically structured care models, limited integration of preventive services, and persistent barriers within the HIV care cascade (including stigma, mental health conditions, limited access to care, and gaps in patient engagement) have been identified as key obstacles to sustained epidemic control, as highlighted by previous research [14,15]. In this context, strengthening integrated, patient-centered health systems that incorporate preventive, clinical, and community-based interventions is essential to improve long-term outcomes and ensure a more comprehensive and sustainable approach to HIV care.

The observed misalignment between HRQoL and healthcare quality indicators can be partially explained by the influence of psychosocial and behavioral determinants. Factors such as treatment adherence, perceived self-efficacy, resilience, and the quality of the patient-provider relationship may buffer the impact of structural limitations in healthcare delivery. In this sense, HRQoL emerges as a multidimensional construct that extends beyond clinical metrics, incorporating subjective and contextual elements that are not fully captured by process-based indicators. While guideline-based indicators such as those proposed by GESIDA are designed to evaluate the quality and excellence of clinical care through standardized processes, they may not fully encompass the complexity of patient-perceived well-being. This conceptual distinction may contribute to the lack of observed correlation between these measures, as highlighted by Nomatshila et al. [16].

Additionally, the prolonged duration of disease and sustained exposure to ART observed in this population suggest a stable trajectory of care, which may further contribute to the preservation of quality of life. As patients achieve long-term virological control, the determinants of HRQoL may shift from predominantly biomedical factors toward psychosocial domains, including mental health, fatigue, chronic symptoms, and social functioning, as described by Skogen et al., who demonstrated that even in well-treated populations, variables such as mental distress, comorbidities, and fatigue play a central role in shaping HRQoL [17].

From an institutional perspective, these findings highlight the need to strengthen integrated care models that go beyond pharmacological treatment and incorporate preventive, educational, and multidisciplinary approaches. The systematic implementation of GESIDA indicators could serve as a valuable tool to identify gaps in care delivery, particularly in high-demand public healthcare settings (such as the referral center evaluated in this study) where high patient volume, the influx of patients from surrounding municipalities, and the episodic nature of care may limit the consistent application of preventive strategies. Addressing these gaps is essential to ensure a comprehensive and sustainable approach to HIV care.

This study has several limitations. Its cross-sectional design precludes establishing causal relationships between HRQoL and healthcare quality indicators. The relatively small sample size and the use of non-probabilistic sampling may limit the generalizability of the findings. Additionally, GESIDA indicators evaluate specific aspects of healthcare processes and may not fully capture the complexity of patient-centered care. Although HRQoL scores showed relatively limited variability, with most participants reporting high values, this likely reflects the clinical stability of the study population rather than a measurement limitation. Nevertheless, a certain degree of range restriction may have reduced the sensitivity to detect subtle correlations between variables.

The results of this study demonstrate that high perceived quality of life can coexist with lower overall compliance with guideline-based healthcare indicators, reflecting a misalignment between objective measures of healthcare quality and patient-reported outcomes. This pattern was characterized by differential performance across domains, with higher compliance in treatment-related indicators and lower performance in preventive and comorbidity-related measures. These findings underscore the importance of adopting a comprehensive, biopsychosocial approach to HIV care, integrating clinical management with preventive strategies and psychosocial support to optimize long-term patient well-being.

## Conclusions

In this study, people living with HIV exhibited a high level of HRQoL despite low compliance with guideline-based preventive care indicators. No significant correlation was observed between these variables, highlighting a misalignment between objective measures of healthcare quality and patient-reported outcomes. These findings suggest that clinical stability and psychosocial factors may play a more relevant role in perceived well-being than structured care indicators alone. Strengthening preventive strategies and integrating a comprehensive, patient-centered approach are essential to optimize long-term outcomes in this population.
